# Disseminated histoplasmosis with conjunctival involvement in an immunocompromised patient

**DOI:** 10.4103/2589-0557.68999

**Published:** 2010

**Authors:** Arun Shirali, Jyoti Kini, Anjith Vupputuri, Maria Kuruvila, M. Venkatraya Prabhu

**Affiliations:** Department of Medicine, Kasturba Medical College, Mangalore, Karnataka, India; 1 Department of Pathology, Kasturba Medical College, Mangalore, Karnataka, India; 2 Department of Dermatology and Venereology, Kasturba Medical College, Mangalore, Karnataka, India

**Keywords:** Acquired immunodeficiency syndrome, conjunctival histoplasmosis, disseminated histoplasmosis, India

## Abstract

We report a case of disseminated histoplasmosis in a 37-year-old male acquired immunodeficiency syndrome patient from south India. The patient presented with high-grade fever, cough, conjunctival nodule and papulonodular hyperpigmented skin lesions. Histology of skin lesions and conjunctival nodule showed numerous intracellular Periodic Acid Schiff-positive rounded yeast cells within macrophages. Bone marrow aspirate confirmed disseminated histoplasmosis. The patient showed dramatic response after starting treatment with Amphotercin B.

## INTRODUCTION

Histoplasmosis refers to a granulomatous infection caused by *Histoplasma capsulatum*, a dimorphic fungus. The fungus has two variants. *H. capsulatum* var. *capsulatum*, which is more ubiquitous, generally causes a subclinical infection. Symptomatic disease presents as a spectrum ranging from chronic progressive pulmonary infection to acute fulminant, or chronic, disseminated histoplasmosis. Infections due to *H. capsulatum* var. *duboisii* are restricted to West Africa. The pulmonary and disseminated forms of histoplasmosis are very common in acquired immunodeficiency syndrome (AIDS) patients. *H. capsulatum* infection is uncommon in India, with only occasional case reports published in the literature. We report one such rare case of disseminated histoplasmosis.

## CASE REPORT

A 37-year-old male farmer, hailing from Puttur, Karnataka, a known case of AIDS on antiretroviral treatment, presented with high-grade fever, cough with expectoration of 6 weeks duration and multiple papulonodular lesions on the face, neck and chest of 2 weeks duration. The lesions were sudden in onset and were gradually progressive.

**Figure 1:**
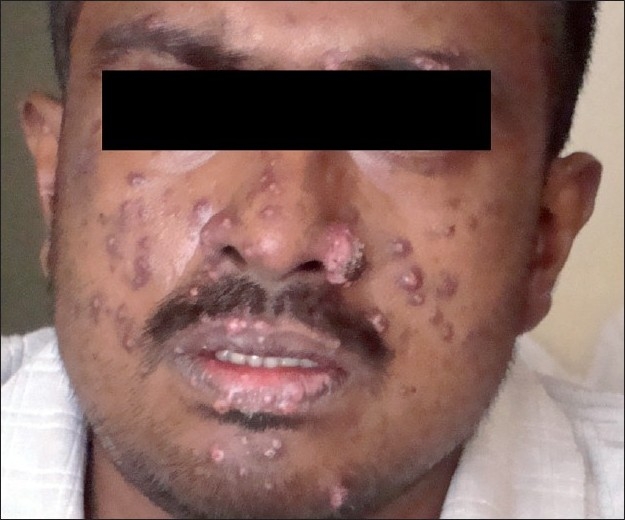
Papulonodular lesions over the face with crusting

**Figure 2:**
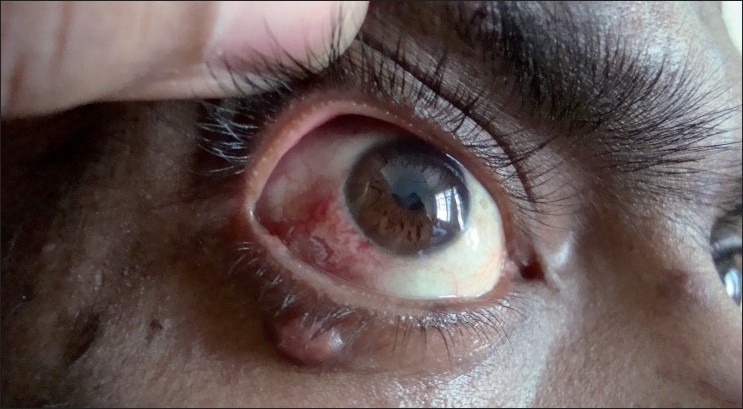
Conjunctival nodule with congestion in the right eye

On examination, he had multiple, discrete, mildly tender, hyperpigmented, papulonodular lesions, distributed over the face [Figure 1], neck, chest, back and oral cavity. Some of them showed ulcerations and crusting. A bright red, elevated, non-mobile, bulbar conjunctival nodule with congestion was present in the right eye [Figure 2]. He had generalized lymphadenopathy and non-tender hepatomegaly. Examination of the respiratory system revealed bilateral coarse crackles. Other systemic examination and fundus examination were unremarkable.

On investigation, his hemoglobin was 6.5 gm/dL, total count was 2220/mm^3^, differential count was neutrophils 81, lymphocytes 15, monocytes 2 and eosinophils 2 and his platelet count was 1.69 lakhs. Peripheral smear showed microcytic hypochromic red blood cells, anisopoikilocytosis, polychromasia and reduced leucocyte count and toxic granules with no evidence of hemolysis. The erythrocyte sedimentation rate was 140 mm/h. The CD4 count was 150/mL. Serum lactate dehydrogenase (LDH) was 842 IU/L. The Venereal Disease Research Laboratory (VDRL) test was non-reactive. Urine and stool examinations were within normal limits. Liver and renal function tests were normal. Blood culture was negative. Chest roentgenogram showed reticulonodular pattern with hilar lymphadenopathy. Sputum examination for *Mycobacterium tuberculosis* was negative.

**Figure 3:**
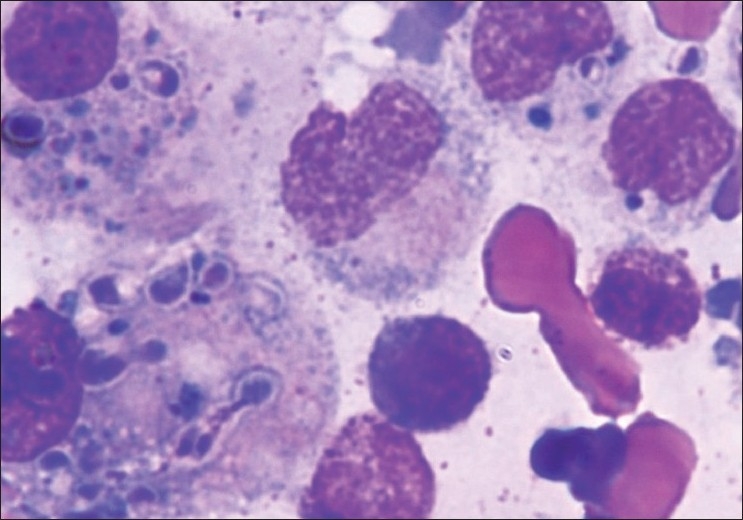
Bone marrow aspirate smears showing a reactive marrow with few extracellular and intracellular histoplasma within macrophages (Leishman stain, ×100)

Leishman-stained bone marrow aspirate smears showed a reactive marrow with few extracellular and intracellular *H. capsulatum* within macrophages [Figure 3].

Skin biopsy specimen showed dermis with aggregates of numerous macrophages containing rounded to oval organisms surrounded by a clear space within the cytoplasm. The organisms were positive for Periodic Acid Schiff (PAS) stain, confirming *H. capsulatum* infection. There was minimal inflammatory response around the macrophages.

**Figure 4:**
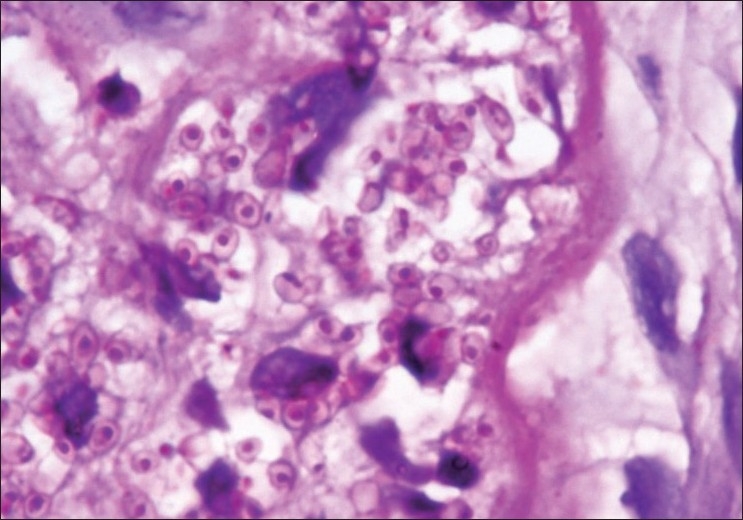
Histopathological section of the conjunctival specimen showing numerous macrophage aggregates with Period Acid Schiff (PAS)-positive histoplasma (PAS stain, ×100)

Histopathological examination of the conjunctival specimen showed numerous macrophage aggregates with 2–4 mm PAS-positive yeast-like forms of *H. capsulatum* [Figure 4].

The patient was started on IV Amphotericin B at a dose of 1 mg/kg/day. Antiretroviral therapy with zidovudine, lamivudine and efavirenz was continued. He became afebrile within 48 h after starting treatment. The skin lesions and the conjunctival lesion healed within 10 days. He was then switched over to oral itraconazole 200 mg twice daily and discharged with an advice to continue the drug for his lifetime.

## DISCUSSION

Histoplasmosis, acquired by inhalation of mycelial fragments and microconidia, is most often self-limiting but can nonetheless cause potentially lethal infection in patients with pre-existing conditions. It remains a frequent cause of opportunistic infection among patients whose immune system is impaired either by pharmaceutical agents or by human immunodeficiency virus (HIV) in endemic areas. Histoplasmosis can present as pulmonary histoplasmosis or progressive disseminated histoplasmosis (PDH). *H. capsulatum* var. *duboisii* presents a natural cutaneous infection, whereas cutaneous dissemination is rare in *H. capsulatum* var. *capsulatum* infection and is mostly associated with AIDS. There are few cases of eye involvement in histoplasmosis reported in the past but isolated conjunctival involvement is very rare and thus far, to the best of our knowledge, there is only one case reported in the literature.[1]

In HIV-infected individuals, the risk factors for the development of histoplasmosis are CD4 count of <200 cells/μL, history of exposure to chicken coops and a known positive serology for complement fixing antibodies prior to illness.[2] Fever and weight loss are found in 90% of the patients with AIDS and PDH. The most common physical findings include rales, hepatosplenomegaly and lymphadenopathy. Mucosal ulcers are not common in the acute form but are common in the subacute and chronic forms. Skin lesions are highly variable, ranging from plaques, papules and nodules with or without crusting. This variability is found to have a significant association with the immune status of the patient (as measured by CD4+ cell count).[3,4] In the absence of history of local trauma, conjunctival involvement is due to hematogenous seeding similar to mucocutaneous loci of infection. Chest X-ray typically demonstrates widely scattered nodular opacities or a diffuse reticular pattern.[5] Serum LDH is elevated in most of the cases and serum LDH >600 IU/L can be used as a diagnostic clue for disseminated histoplasmosis.[6,7]

Histoplasmosis is sporadically reported from India and is considered to be endemic in certain areas of East India,[8] but recent case reports from south India and a retrospective study by Subramanian *et al*. indicate that histoplasmosis has no particular predilection to East India.[9,10] Our patient had the risk factors of exposure to soil and AIDS with CD4 count of 150 cells/μL. He also had clinical features and investigations consistent with histoplasmosis as described in the literature, but our case is unique from the remainder of the reported cases as it has extensive involvement of skin, bone marrow and conjunctiva, confirmed on microscopic examination. The conjunctival lesion was similar to the mucocutaneous lesions seen in patients with systemic histoplasmosis. A further culture or immunofluorescence could not be performed for definitive identification and subtyping due to financial constraints. However, the dramatic response to Amphotercin B, characterized by improvement of skin lesions and the patient becoming afebrile, cannot be overemphasized.

Thus, in the current scenario of AIDS pandemic, a high index of suspicion has to be maintained in diagnosing rare opportunistic infections like histoplasmosis even in non-endemic parts of the world. Serum LDH can be used as a diagnostic clue. A timely diagnosis and appropriate medical intervention results in a good prognosis.
